# Convergent evolution of skin surface microarchitecture and increased skin hydrophobicity in semi-aquatic anole lizards

**DOI:** 10.1242/jeb.242939

**Published:** 2021-10-14

**Authors:** Simon Baeckens, Marie Temmerman, Stanislav N. Gorb, Chiara Neto, Martin J. Whiting, Raoul Van Damme

**Affiliations:** 1 Laboratory for Functional Morphology, Department of Biology, University of Antwerp, 2610 Wilrijk, Belgium; 2 Department of Biological Sciences, Macquarie University, Sydney, NSW 2109, Australia; 3 Laboratory for the Evolution and Optics of Nanostructures, Department of Biology, Ghent University, 9000 Gent, Belgium; 4 Functional Morphology and Biomechanics, Zoological Institute of the Christian Albrecht Universität zu Kiel, Am Botanischen Garten 9, 24118 Kiel, Germany; 5 School of Chemistry and Sydney Nano Institute, The University of Sydney, Sydney, NSW 2006, Australia

**Keywords:** *Anolis*, Functional surfaces, Non-wettability, Squamate integument

## Abstract

Animals that habitually cross the boundary between water and land face specific challenges with respect to locomotion, respiration, insulation, fouling and waterproofing. Many semi-aquatic invertebrates and plants have developed complex surface microstructures with water-repellent properties to overcome these problems, but equivalent adaptations of the skin have not been reported for vertebrates that encounter similar environmental challenges. Here, we document the first evidence of evolutionary convergence of hydrophobic structured skin in a group of semi-aquatic tetrapods. We show that the skin surface of semi-aquatic species of *Anolis* lizards is characterized by a more elaborate microstructural architecture (i.e. longer spines and spinules) and a lower wettability relative to closely related terrestrial species. In addition, phylogenetic comparative models reveal repeated independent evolution of enhanced skin hydrophobicity associated with the transition to a semi-aquatic lifestyle, providing evidence of adaptation. Our findings invite a new and exciting line of inquiry into the ecological significance, evolutionary origin and developmental basis of hydrophobic skin surfaces in semi-aquatic lizards, which is essential for understanding why and how the observed skin adaptations evolved in some and not other semi-aquatic tetrapod lineages.

## Abstract (precis)

**Summary:** Multiple *Anolis* lineages independently evolved a similar skin surface microarchitecture with water-repellent properties as an adaptation to a semi-aquatic lifestyle.

## INTRODUCTION

Terrestrial animals that venture into the water on a regular basis face a number of challenges not encountered by their strictly terrestrial counterparts. While submerged, they must deal with hydrodynamic drag forces hindering locomotion and with the risk of running out of air (Fish, 1993; Webb, 1988). Back on land, the film of water adhering to their body surface may interfere with locomotion (Haldane, 1956) and thermoregulation (Kerslake and Waddell, 1958; Webb and King, 1984) or may increase the risk of biofouling (Railkin, 2020). Various semi-aquatic invertebrates and plants have evolved skin adaptations in the form of surface microstructures that increase the hydrophobic nature of the skin surface (Barthlott et al., 2016). These hydrophobic surfaces can capture and retain a thin layer of air while submerged in water, which enables underwater breathing and reduces fluid drag in insects (Ditsche-Kuru et al., 2011; Flynn and Bush, 2008); they also facilitate self-cleaning by removing contaminating particles on the skin as water droplets roll over the surface (Barthlott and Neinhuis, 1997; Blossey, 2003). Multiple tetrapod lineages have secondarily adopted a semi-aquatic lifestyle and have reshaped aspects of their phenotype accordingly and convergently (Houssaye and Fish, 2016). Intriguingly, it has never been documented that this would include structural modification of the integument, resulting in increased hydrophobicity.

**Fig. 1.**
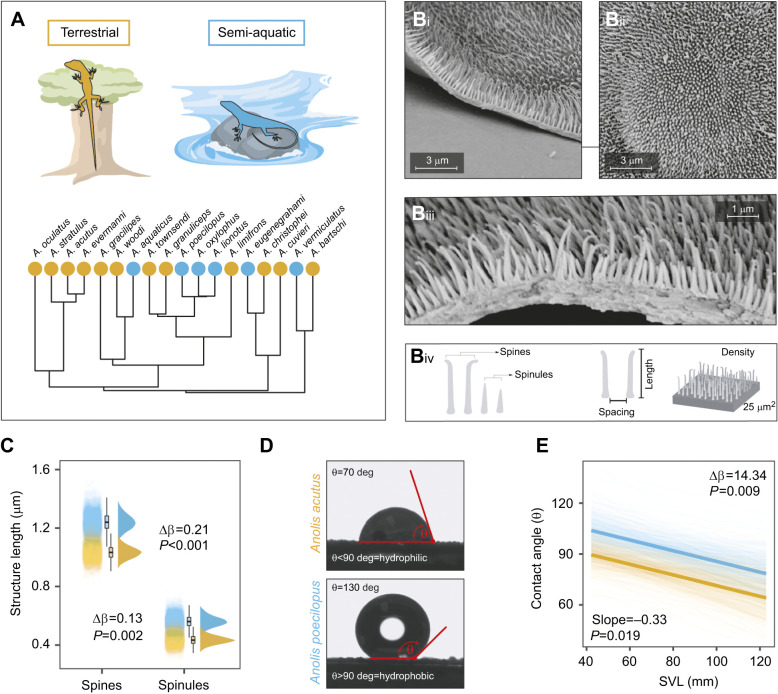
**Multiple *Anolis* lineages independently evolved similar skin surface microarchitecture with water-repellent properties as an adaptation to semi-aquatic life.** (A) Phylogeny of the *Anolis* species under study. Colors denote species lifestyle (orange, terrestrial; blue, semi-aquatic). (B) Typical scanning electron micrographs of *Anolis* skin surface architecture (i and iii, side view; ii, top view) and (iv) a graphical illustration of the epidermal outgrowths with annotations on the structural traits of interest. (C) The posterior distribution of model predictions for spine and spinule length shows the structural surface differences between semi-aquatic and terrestrial anoles. (D) Examples of a low (top; *A. acutus*) and high (bottom; *A. poecilopus*) water contact angle value indicating a hydrophilic and hydrophobic skin surface, respectively. Note that the white center in the bottom droplet is merely the result of light reflection. (E) The posterior distribution of model predictions for contact angle value against body size indicates that, irrespective of size, the skin surface of semi-aquatic anoles is more hydrophobic than that of terrestrial anoles (thick lines represent posterior means). SVL, snout–vent length.

The transition to a semi-aquatic lifestyle has independently occurred multiple times throughout the evolutionary history of the lizard genus *Anolis* (Leal et al., 2002; Losos, 2009; Muñoz et al., 2015) (Fig. 1A). In anoles, the skin surface is covered with microscopic hair-like ornaments (Peterson, 1984), and contingent upon its complexity, organization and length dimensions, these hair-like microstructures may have the potential to generate extreme surface hydrophobicity (Gao et al., 2011; Li and Amirfazli, 2008; Yan et al., 2011). Indeed, similar skin surface microstructures have been found in gekkonid lizards and shown to be responsible for the highly hydrophobic surface of gecko skin (Riedel et al., 2020; Watson et al., 2010, 2015a). The water-resistant properties of anole skin, however, have remained unexamined, but very recent discoveries have provided some insight into this matter. Researchers observed that semi-aquatic *Anolis* lizards are able to sustain long periods submerged underwater by iteratively expiring and re-inspiring narial air bubbles (Boccia et al., 2021). As in semi-aquatic insects, a hydrophobic skin is a key requirement for the underwater formation of an air bubble – hence, functional respiration (Flynn and Bush, 2008; Shirtcliffe et al., 2006) – so a hydrophobic skin in semi-aquatic anoles is implied. However, whether a hydrophobic structured skin surface in anoles has evolved in response to life at the water–land interface is still an open question. Answering this question is the primary goal of our study.

The microscopic hair-like ornaments on the *Anolis* skin surface are a plesiomorphic trait acquired from the anoline ancestor (Peterson, 1983a, 1984; Peterson and Williams, 1981). Although the original selective advantage of such an ornamented skin surface in the anoline ancestor, or why hair-like structures originated in the first place, remains somewhat equivocal, a possible role in facilitating ecdysis has received most attention (Alibardi and Maderson, 2003; Bauer, 2019; Irish et al., 1988; Maderson, 1970). As such, we hypothesize that the pre-existing surface architecture of anoles may have acted as a San Marconian spandrel (Gould and Lewontin, 1979) in semi-aquatic species by serving as a basis for the development of a more elaborate surface structural organization with high hydrophobic properties. To examine this, we compared the microstructural architecture and wettability of the skin surface of semi-aquatic and closely related terrestrial anole species, and tested for evolutionary convergence of skin form and function.

## MATERIALS AND METHODS

### Specimens

We examined 52 well-preserved specimens representing 18 *Anolis* species, six of which are generally considered ‘semi-aquatic’ (Leal et al., 2002; Losos, 2009; Muñoz et al., 2015) because they inhabit riparian habitats, and systematically enter the water to escape predators or in search of prey (Birt et al., 2001; Campbell, 1973; Eifler and Eifler, 2010; Flaschendräger, 1992; Herrmann, 2017; Miyata, 1985; Ream and Reider, 2013; Rodríguez-Schettino and Novo Rodríguez, 1985; Rodríguez-Schettino et al., 1987; Schwartz, 1978; Swierk, 2019; Vitt et al., 1995; Williams, 1984). Behavioral observation indicate that semi-aquatic species can remain under water for up to 18 min (Boccia et al., 2021). Of the six semi-aquatic species included in this study, four inhabit Central America (*A. aquaticus*, *A. lionotus*, *A. oxylophus* and *A. poecilopus*) and two inhabit the Caribbean (*A. eugenegrahami* from Hispaniola and *A. vermiculatus* from Cuba). Based on availability, destructive sampling permission and phylogenic proximity (Poe et al., 2017), we supplemented our dataset with 12 terrestrial species (*A. oculatus*, *A. stratulus*, *A. acutus*, *A. evermanni*, *A. gracilipes*, *A. woodi*, *A. townsendi*, *A. granuliceps*, *A. limifrons*, *A. christophei*, *A. cuvieri* and *A. bartschi*). Phylogenetic reconstructions suggest a total of six independent transitions to a semi-aquatic lifestyle within the genus *Anolis* (Herrmann, 2017; Muñoz et al., 2015; Poe et al., 2017)*.* Our study covers at least four of these, allowing tests of convergence.

Fifty-two specimens (2–6 per species) preserved in ethanol were obtained from the herpetological collections of five American natural history museums [American Museum of Natural History (AMNH), Natural History Museum of Los Angeles County (LACM), Museum of Comparative Zoology (MCZ), University of Florida (UF), University of Kansas Museum of Natural History (UKMNH); Table S1]. Liquid-preserved specimens are widely used for the study of skin surface ornamentation in squamates because the keratinized oberhautchen layer of the skin surface preserves well (Harvey, 1993; Irish et al., 1988; Martinez et al., 2021; Matveyeva and Ananjeva, 1995; Peterson, 1984; Riedel et al., 2015; Ruibal, 1968). Previous work has found no significant effect of preservation on anole skin surface structure (Baeckens et al., 2019). In order to eliminate any potential effect of ontogenetic or intersexual variation in skin surface anatomy and to increase statistical power, only male adult lizards were included in this study. Prior to imaging and wettability tests of the lizards’ skin surface, snout-to-vent length (SVL) of each individual was measured using digital calipers (Mitutuyo; precision 0.01 mm).

### Skin surface preparation

From each specimen, we excised one patch of skin (approximately 1×2 cm) at the same body region on the flank (dorsolateral), i.e. posterior to the midpoint between the pectoral and pelvic girdle – same anatomical location as in Baeckens et al. (2019). Although the dorsolateral flank of most species in our study is dull green or brown, we made sure to avoid excising highly conspicuous and vivid color badges. Following standard protocols of studies of lizard skin surface microstructure (Harvey, 1993; Irish et al., 1988; Matveyeva and Ananjeva, 1995; Peterson, 1984; Riedel et al., 2015; Ruibal, 1968), excised samples were lightly brushed with a fine paintbrush to remove any surface debris, dehydrated in a graded ethanol series, and critical point dried (Leica EM CPD300; 20 exchanges, medium heat with vent). Skin patches were then cut into two similar-sized patches (approximately 1×1 cm), of which one skin patch was used for imaging and quantifying skin surface microstructure, and one for assessing skin surface wettability by contact angle goniometry.

### Skin surface imaging

Prior to imaging, dried skin patches were individually affixed to aluminium stubs using double-sided carbon conductive tape and sputter coated (Emitech K550) with a 20 nm-thick gold layer (2 min deposit at 20 mA). All samples were then studied in a scanning electron microscope (Phenom XL SEM) operating at an accelerating voltage of 10 kV (full backscatter detector) with a 1 Pa vacuum level and maximal image resolution (2048×2048 pixels). A total of approximately 1000 images were taken at magnifications between ×185 and ×20,000.

### Quantifying skin surface structure

The skin surface of anoles consists of granular scales covered by epidermal fibrillar outgrowths (Peterson, 1983a, 1984; Peterson and Williams, 1981). Explorative visual assessment showed that the anole skin surface is covered by fine structures of varying height consisting of two distinct lengths: short, tapered outgrowths (which we call here ‘spinules’) and elongated outgrowths with a slightly hooked end (which we call here ‘spines’). Terminology classification is consistent with Garner and Russell (2021). The following variables of the surface structure of anole skin were extracted from the SEM images and quantified using ImageJ (Abràmof et al., 2005): (1) scale area (mm^2^) – the area of 10 scales per skin sample; (2) structure density – the number of hair-like structures (irrespective of length category) per 25 µm^2^, assessed on average 5 times (each from a different location on the skin) per skin sample; (3) structure length (µm) – the length of the hair-like structures from the base to the very tip (15 spines and 15 spinules measured per skin sample) and; (4) structure spacing (µm) – the distance between the bases of two adjacent structures (irrespective of length category), measured 10 times per skin sample. Surface structure was quantified blindly, i.e. the species name and habitat category were kept unknown to the researcher (M.T.).

### Assessing surface wettability with contact angle goniometry

Wettability, the process of water interacting with a surface, is commonly characterized by the contact angle (θ), which is defined as the angle between the tangent to the liquid–vapor interface and the solid surface at the three-phase contact line; by convention, the contact angle is measured from the liquid side (Bangham and Razouk, 1937; Baxter, 1950; Huhtamäki et al., 2018; Öner and McCarthy, 2000; Wenzel, 1936). The equilibrium contact angle is obtained when the liquid's cohesion forces balance the liquid–solid and the solid–vapor adhesion forces. Low contact angle values demonstrate a tendency of the water to spread and adhere to the surface, whereas high contact angles show the surface's tendency to repel water (Baxter, 1950; Fox and Zisman, 1950; Owens and Wendt, 1969; Wenzel, 1936). A surface is considered hydrophilic when the contact angle is below 90 deg and hydrophobic when the angle is over 90 deg (Quéré, 2008; Zhao and Jiang, 2018). While there are various well-developed conventional methods for measuring contact angle (Carrier and Bonn, 2015; Mittal, 2020; Neumann and Good, 1979; Żenkiewicz, 2007; Zhao and Jiang, 2018), sessile-drop goniometry is the most commonly used (Drelich, 2013; Huhtamäki et al., 2018; Kwok and Neumann, 1999; Schuster et al., 2015; Zhao and Jiang, 2018). Briefly, a video recording captures the side-on profile of a water drop on a solid surface and the contact angle is determined from the images of the video by a manual or automatic fitting procedure. In our study, we determined the ‘static contact angle’, which is the contact angle of a stationary water drop that was deposited on the skin surface. Because a deposited drop is not necessarily in an equilibrium state of absolute minimal total free energy (Baxter, 1950; Chibowski and Perea-Carpio, 2002; Eral et al., 2013), repeated measurements per sample are recommended (Neumann and Good, 1979). Therefore, we repeated the procedure for up to 9 different locations on each skin sample (mean±s.e.m. 4.3±0.3), which translated to up to 31 deposited drops per species (11.8±1.8).

#### Experimental set-up and design

We followed the guidelines for surface-wetting characterization using contact angle measurements by Huhtamäki et al. (2018), Schuster et al. (2015) and Zhao and Jiang (2018). In our study, wetting behavior assessment via the sessile drop technique was carried out using a Krüss DSA30S goniometer and associated DSA4 drop shape analysis software (Krüss GmbH, Hamburg, Germany). The goniometer was set on a sturdy table in a room with a constant temperature of 23±0.5°C (mean±s.e.m.). First, the cleaned glass syringe (500 µl with Luer lock connector) connected to the liquid dispensing system was filled with Milli-Q^®^ water. Second, an individual dried skin sample was mounted on aluminium stubs using double-sided carbon conductive tape and placed macroscopically flat and rigid in a stub-holder on the goniometer sample stage. Dried skin samples were never touched with bare fingers (gloves were always worn), but were handled with dry ethanol-cleaned forceps to prevent skin fouling. Third, we made sure that the camera view was on the same plane as the sample. Camera settings were tweaked as recommended by Huhtamäki et al. (2018). Fourth, the motorized syringe dispenser was lowered so that the tip of the needle (steel; 0.5 mm diameter) was in focus in the top quartile of the video frame. The disposable needle was replaced prior to the start of each measurement. Next, a ∼6 µl droplet was automatically dispensed so that it freely hung on the tip of the needle. The sample stage was then slowly and steadily raised until the droplet came into contact with the skin sample, and subsequently gently lowered so the drop got detached from the needle. The overall procedure was videotaped (1 frame s^−1^) and repeated on multiple locations per skin sample.

#### Contact angle quantification

Images of the sessile-drop videos were imported and analyzed in ImageJ (Abràmof et al., 2005). Per video, contact angles were quantified manually on two different images. Angles were quantified blindly, i.e. the species name and habitat category were kept unknown to the researcher (M.T.). Contact angle measurements were repeated 6 times per image (or ‘video frame’): 3 times on the left-hand side and 3 times on the right-hand side of the deposited drop. With an overall total of 2490 measurements, we acquired on average 50±4 (range 12–108) measurements per specimen from multiple wettability tests. In ImageJ, the ‘contact angle plug-in’ enables the automatic calculation of the contact angle. The plug-in allows more sophisticated mathematical curve fitting using a circle and ellipse approximation, which generates the following output: angle based on straight-line analysis, angle based on the best-fit circle, and angle based on the best-fit ellipse. (For more details on the automatic contact angle calculations, see https://imagej.nih.gov/ij/plugins/contact-angle.html.) Although labor intensive, manual angle analysis is typically recommended over automatic fitting procedures as the latter can sometimes fail and generate outliers (Huhtamäki et al., 2018). To be thorough, we also quantified contact angles based on the three aforementioned automatic approaches. In total, 4240 contact angle measurements were taken and all four quantification approaches provided highly intercorrelated contact angles (interclass correlation, ICC=0.93, *P*<0.001; Koo and Li, 2016). All further statistical analyses were based on the manual contact angle analysis.

### Statistics

All data were statistically analyzed in R version 3.6. To test whether skin surface structure and wettability differ between semi-aquatic and terrestrial anole species, we used Monte Carlo Markov chain generalized linear mixed models (‘MCMCglmm’ package; Hadfield, 2010) as this enables the inclusion of a phylogenetic structure in a Bayesian generalized linear modeling framework (Hadfield, 2010; Hadfield and Nakagawa, 2010). The recent phylogenetic tree proposed by Poe et al. (2017) was pruned to include only the 18 species implemented in this study. In a first series of model fitting, we tested for each skin surface structure variable separately (i.e. scale area, spine length, spinule length, structure density, structure spacing) to see whether variation in structure (response variable) could be explained by species habitat. In each model, we included ‘habitat’ (two-level factor: semi-aquatic versus terrestrial) and SVL (covariate) as fixed effects; SVL was included to account for a potential scaling effect of skin surface morphology (e.g. Webster et al., 2009). We used Gaussian models with ‘phylogeny’, ‘SEM image’ and ‘individual’ (nested in ‘species’) as random effects to account for repeated measures. These models revealed that none of the microstructural traits (scale area excluded) were significantly affected by SVL (slope with SVL: spine, α=0.002, credible interval CI=[−0.001, 0.003], pMCMC=0.110; spinule, α=0.003, CI=[−0.001, 0.006], pMCMC=0.066; spacing, α=0.001, CI=[−0.001, 0.003], pMCMC=0.235; density, α=−0.988, CI=[−2.382, 0.506], pMCMC=0.197). Therefore, we repeated the models but solely with ‘habitat’ as fixed effect. Note that in this study there was no significant difference in SVL between terrestrial and semi-aquatic anole lizards (β=−16.65, CI=[−35.80, 1.89], pMCMC=0.080). In a second series of model fitting, we tested the explanatory power of species habitat on variation in contact angle (θ) as a measure for skin surface wettability. Fixed effects were ‘habitat’ and ‘SVL’. Random effects were ‘phylogeny’, ‘individual’ (nested in ‘species’), ‘video recording’, ‘video frame of analysis’ and ‘position of measurements’ (two-level factor: left- versus right-hand side of the drop). Thirdly, we explored the direct link between surface structure design and wettability. Based on species averages, we ran a model with contact angle as response variable, all structural skin surface variables as fixed variables, and phylogeny as random. To determine which structural trait(s) explained most of the variation in contact angle, we used the ‘MuMIn’ package (https://cran.r-project.org/web/packages/MuMIn/MuMIn.pdf) for automated model selection by subsetting of the maximal model; model parameter and prediction averaging were based on model weights derived from information criteria averages (deviance information criterion, DIC). In all Bayesian models, we used an inverse-Wishart prior (*V*=1, *v*=0.002) for both the residual term and the random effect. Each model was run for 5,000,000 iterations with a 1000 burn-in. Chains were sampled every 500 iterations. Default model parameters were chosen based on the recommendations of Hadfield (2021) and Garamszegi (2014). From the models, we calculated the posterior mode and 95% credible interval (CI) for the intercept (β) to assess whether the response variable significantly differs between semi-aquatic and terrestrial species.

To investigate whether and how changes in lifestyle may have influenced the evolution of skin surface structure and wettability, we used an evolutionary model selection framework. These models require one data entry per species, so species averages were calculated for each trait. We were particularly interested in those traits that preceding Bayesian generalized linear models indicated to vary among semi-aquatic and terrestrial species. We tested three different models of evolution using the methods and codes (‘ouch’ package) developed by Butler and King (2004). Out of the three models, the first model tested whether the trait of interest varies at random following a Brownian motion (BM) process, where phenotypic variation accumulates with time. A rejection of the BM model implies that phenotypic evolution has not followed a random evolutionary trajectory (neutral drift). The two other models followed an Ornstein–Uhlenbeck (OU) process (Cressler et al., 2015; Hansen, 1997; Lande, 1976), with the simplest model (‘OU1’) having a single optimal for all species regardless of selective regime. A third model (‘OU2’) adds additional optima for each selective regime so that we have separate optima for the two different habitat types (i.e. semi-aquatic and terrestrial) by estimating an ancestral regime optimum for all internal branches (based on maximum likelihood). To determine the goodness of fit of candidate evolutionary models, we compared the likelihood of the models by means of a chi-square test.

## RESULTS

Scanning electron microscopy revealed that the (dorsolateral) skin surface of both semi-aquatic and terrestrial *Anolis* species contained scales that were densely covered with hair-like structures, consisting of interspersed spines and spinules (Fig. 1B). The granular scales of the anole species under study were non-overlapping and dome shaped, with sizes ranging from 0.01 to 0.24 mm^2^ in surface area. Spines were roughly 1 μm in length, about twice as long as spinules, and had tapered, blunt ends. Unlike spinules, most spines showed a slight curving at their top ends. The density of spines and spinules was high, ranging from 150 up to 350 hairs per 5×5 µm^2^ square grid, resulting in a submicrometer spacing (range 0.09–0.23 µm) of these hairs (Table 1). Bayesian generalized linear mixed models indicated that both spines and spinules were significantly longer in semi-aquatic anole species compared with terrestrial species (spinules, β=0.13, CI=[0.05, 0.21], pMCMC=0.002; spines, β=0.21, CI=[0.08, 0.33], pMCMC<0.001; Fig. 1C, Table 1). The length difference between spines and spinules was not related to lifestyle (β=0.09, CI=[−0.10, 0.28], pMCMC=0.321). Our models showed no difference in structural spacing or density between semi-aquatic and terrestrial species (spacing, β=0.02, CI=[−0.05, 0.09], pMCMC=0.636; density, β=17, CI=[−76, 43], pMCMC=0.562; Table 1). Scale area increased with body size (α=1.65, CI=[1.01, 2.28], pMCMC<0.001), but was unaffected by species lifestyle (β=23.01, CI=[−8.47, 52.36], pMCMC=0.130).

**Table 1.**
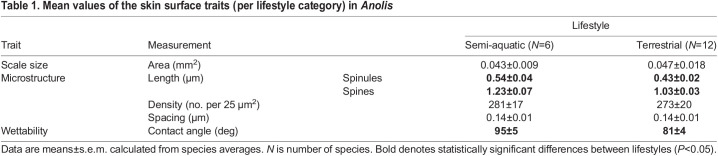
Mean values of the skin surface traits (per lifestyle category) in *Anolis*

We examined skin surface wettability using contact angle goniometry (Fig. 1D), whereby surfaces with water contact angles greater than 90 deg are considered hydrophobic (Zhao and Jiang, 2018). The mean±s.e.m. species contact angle ranged from 59±1 deg in *A. cuvieri* up to 120±18 deg in *A. eugenegrahami*. Contact angle slightly decreased with body size (slope=−0.33, CI=[−0.64, −0.06], pMCMC=0.019) and varied between species occupying different habitats: semi-aquatic anoles had significantly greater contact angle values than terrestrial species (intercept difference: Δβ=14.34, CI=[3.57, 26.02], pMCMC=0.009; Fig. 1E).

Automated selection of the model explaining most of the variation in contact angle indicated that the best-fitting model with the highest support (d.f.=6, log likelihood=−12.86, DIC=−18.1, ΔDIC=0.0, weight=0.684) included three structural variables: spine length (coefficient=42.64), scale area (coefficient=−28.81) and spacing (coefficient=190.60). The second-best model also showed high support (d.f.=7, log likelihood =−16.75, DIC=−15.3, ΔDIC=2.85, weight=0.164) and was similar to the first model but included also spinule length (coefficient=23.80), aside from spine length (coefficient=28.38), scale area (coefficient=−29.21) and spacing (coefficient=207.30). Overall, the surface structures that explained most of the variation in contact angle values were spine and spinule length, spacing and scale area.

In our models of trait evolution, we focused on spine length, spinule length and contact angle because these were the traits that varied significantly between semi- and non-aquatic anoles. When fitting models of trait evolution, the adaptive model OU(2) was shown to be the best-fitting model for all three traits, receiving more support than the BM or the simplest adaptive model, OU(1), having a single optimal for all species regardless of selective regime (Table 2). These results suggest that the evolution of skin surface structure (spine and spinule length) and surface wettability (contact angle) oscillates round two phenotypic optima, one for each selective regime (i.e. semi-aquatic and terrestrial).

**Table 2.**
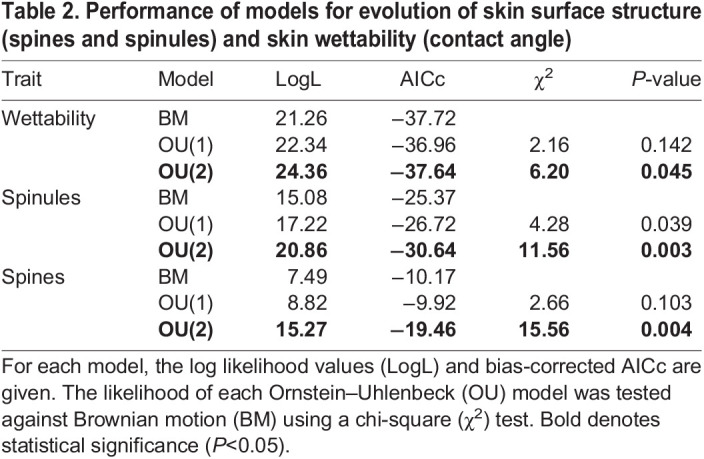
Performance of models for evolution of skin surface structure (spines and spinules) and skin wettability (contact angle)

## DISCUSSION

### The spinulate surface microarchitecture of anoles

Consistent with the assessments by Ruibal (1968) and Peterson (1983a,b, 1984), we observed that the skin surface of anole lizards is densely covered by hair-like microstructures. Our detailed SEM examination also revealed that there is substantial differentiation in the size and shape of these hair-like microstructures. Specifically, we documented two types of morphologically distinct structures on the skin surface of anoles: short, tapered epidermal fibrillar outgrowths (spinules) and the roughly twice-as-long, more elongated outgrowths with a slightly hooked, blunt end (spines) (Fig. 1B). Moreover, we found substantial variation in spine and spinule length among species, which could be (at least partly) explained by variation in species lifestyle: semi-aquatic anoles carried spines and spinules that were roughly one-quarter longer than those of closely related terrestrial species (Table 1). Earlier work on a small number of *Anolis* species failed to find any correlation between the characteristics of a spinulate surface and a particular habitat or locomotor behavior, but overlooked semi-aquatic anoles (Peterson, 1984). A recent study by Riedel et al. (2019) examined the ecological associates of surface microstructure diversity in terrestrial geckos – there are no semi-aquatic geckos (Bauer and Jackman, 2008) – and found that ground-dwelling species tend to have slightly longer spinules than species inhabiting saxicolous or arboreal environments. Additional data on the microstructure of semi-aquatic species from iguanid lineages other than anoles would help to assess the generality of our findings.

The spinulate microarchitecture of anoles is traditionally viewed as a plesiomorphic character because of its homologies with leiosaurs, or ‘pre-anolines’ (Peterson, 1983a, 1984) – a more basal clade within the Pleurodonta (Pyron, 2017). This view, however, is challenged by the uncertain internal relationships of pleurodont iguanians (Pyron, 2017; Pyron et al., 2013; Wiens and Lambert, 2018), which continues to be a major obstacle in squamate phylogenetics (Simões and Pyron, 2021). The *Anolis* skin surface design closely resembles that of geckos (Riedel et al., 2019; Stewart and Daniel, 1975) and evidence points towards the independent evolution of a spinulate skin surface in iguanid and gekkotan lineages (Bauer, 2019; Peterson, 1984). Spinules are often considered a key morphological innovation because they constitute a necessary prerequisite for the subsequent elaboration of a functional adhesive mechanism – the toepad – through the formation of subdigital setae (Bauer, 2019; Ernst and Ruibal, 1966); setae only take on an adhesive function when they reach a certain length and form and begin to be modified from the more simple spinulate architecture (Russell and Garner, 2021). Yet, the possible selective advantage(s) of a simple spinulate microarchitecture in the anoline ancestor, or why spinules originated in the first place, remains poorly understood. Earlier functional histological research suggests that a spinulate oberhautchen in the common ancestor of anoles might have played an important role in facilitating ecdysis (Alibardi and Maderson, 2003; Bauer, 2019; Irish et al., 1988; Maderson, 1970). Other hypotheses include functions such as self-cleaning and anti-fouling, reduction of friction and wear protection, as has been shown in geckos (Riedel et al., 2020; Spinner et al., 2013; Watson et al., 2015a,b). To fill this gap, future studies on the functional and ecological significance of spinules should explore the more basal iguanid and gekkotan lineages and focus on species that carry simple spinulate microarchitecture but lack setae and functional toepads.

### Hydrophobic hierarchically structured surfaces

The structural organization and dimension of surface ornamentation greatly determine surface wettability (Gao et al., 2011; Li and Amirfazli, 2008; Yan et al., 2011). Surfaces can reach extreme non-wettability by reducing surface contact area through ‘hierarchical structuring’, i.e. multi-scale roughening on the microscale and nanoscale generated by multiple superimposed structural levels (Barthlott et al., 2016). Our SEM images suggest that the skin surface of anoles exhibits a two-level hierarchical structuring consisting of granular scales (primary level of >1 µm) superimposed with dual-scale hair-like structures (secondary level of ≤1 µm), i.e. long spines and short spinules. While such surface structuring suggests high non-wettability potential, our experiments revealed considerable among-species variation in the degree of skin surface wettability, with contact angle values ranging from roughly 60 deg (wettable surface) in the terrestrial *A. cuvieri*, up to 120 deg (highly non-wettable surface) in the semi-aquatic *A. eugenegrahami.* As hypothesized, much of this variation could be explained by anole lifestyle: the skin surface of semi-aquatic species was more hydrophobic (i.e. exhibiting higher contact angle values) than those of strictly terrestrial species. This makes sense, as semi-aquatic anoles carry longer spinules, and a higher spinule length can support a higher proportion of trapped gas and a more stable hydrophobic state (Scarratt et al., 2017). Spinule length also strongly determines surface non-wettability of gecko skin (Riedel et al., 2020) as does the height of micro-protrusions on the wings of cicadas (Sun et al., 2012).

Interestingly, our findings also revealed a minor, but significant, effect of lizard body size on contact angle variation (Fig. 1E). This outcome is most likely the result of body size-driven variation in the proportional size relationship among skin surface microstructures. Because scale size, but not spine and spinule length, increases with body size, the size ratio between the scales and the hair-like microstructures that cover the scales varies across different-sized anoles. Based on the scaling relationship between the area and height of *Anolis* scales (Baeckens et al., 2019), we estimate the ratio of scale height to spinule height to range from circa 25:1 in small-sized anoles up to 80:1 in large-sized anoles. Knowing that the size ratio of two superimposed structural levels can influence surface wettability (Li and Amirfazli, 2008; Patankar, 2004), it is plausible that the skin surfaces of different-sized anoles vary in their wettability properties as a result of differences in the aspect ratio of their microstructures. For comparison, ratios between the micro- and nano-scale protrusions on the surfaces of gecko skin and lotus leaves are roughly 13:1 and 15:1, respectively; these surfaces are termed superhydrophobic, showing contact angle values over 150 deg (Bhushan and Jung, 2011; Feng et al., 2002; Watson et al., 2015a). Based on biophysical theory and natural observations, material scientists consider 10:1 the ‘optimal’ aspect ratio for achieving extreme non-wettability; they fabricate superhydrophobic surfaces covered with ‘raspberry-like particles’, which are nano-sized secondary spheres superimposed on primary particles 10 times that size (D'Acunzi et al., 2010; Telford et al., 2013). So, the observed negative relationship between contact angle and anole body size in this study is likely a scaling effect, a mere consequence of the microstructure ratio of anoles approaching 10:1 with decreasing body size. Indeed, our statistical models showed that both spinule length and scale size explain a large portion of the variation in contact angle, with longer spinules and smaller scales positively influencing skin surface hydrophobicity; Riedel et al. (2020) found the exact same form–function relationship in Australian geckos.

Although hierarchical surface structuring is fundamental for high hydrophobicity in most biological systems, the chemical composition of the surface coating contributes strongly also (Barthlott et al., 2016). The elytra of Namib desert beetles (*Stenocara* sp.), for instance, bears hydrophilic bumps on a waxy hydrophobic coating thought to increase the collection of water from early-morning fog (Parker and Lawrence, 2001). The feathers of ducks combine structural and chemical elements to create a highly water-repellent surface that keeps their body dry (Bakken et al., 2006; Liu et al., 2008). Lipophilic waxes are the most common low surface energy chemicals found on the surfaces of tetrapods (Barthlott et al., 2016), but have not been documented on *Anolis* skin. In fact, the outermost layer of the reptilian epidermis is composed of proteinaceous β-keratin (Alibardi and Toni, 2006; Toni et al., 2007), which is by nature rather hydrophilic (Autumn and Hansen, 2006; Barthlott et al., 2016; Bormashenko et al., 2007; Spinner et al., 2014). Although we cannot rule out the possibility that the skin surface of (some) anoles could be layered with a waxy film, the potential presence (or absence) of skin waxes will not have influenced the outcome of our wettability experiments. Any lipophilic substance that might have covered the skin samples used in our study would have been removed during sample preparation by treating the skin with ethanol, an organic solvent (see Barthlott et al., 2016). As such, the observed variation in surface wettability in this study can be directly and solely accredited to variation in the structural surface complexity of anole skin. Future studies should establish whether the wettability of anole skin is further influenced by chemical surface coatings.

### The ecological need for a hydrophobic skin

Our findings reveal that semi-aquatic anole species repeatedly evolved a hydrophobic structured skin surface, yet, the ultimate question remains unanswered: why? We expect that a hydrophobic skin may benefit semi-aquatic anoles in multiple ways. First and foremost, it may enable ‘plastron respiration’ by capturing and retaining an air film (termed plastron) in the water that can be used as an underwater oxygen supply (Flynn and Bush, 2008; Shirtcliffe et al., 2006). Plastron breathing is well known from several semi- and full-aquatic arthropods (Balmert et al., 2011; Ege, 1915; Kovalev et al., 2020; Thorpe, 1950), but had not been described in a vertebrate species until Boccia and colleagues (2021) recently discovered it in several anoles. Boccia et al. (2021) report that semi-aquatic anoles rebreathe underwater via recurrent inhalation and exhalation of an air bubble around the lizard's nostrils. The researchers showed, furthermore, that such sustained underwater rebreathing evolved convergently in semi-aquatic anole species. Anole ‘scuba-diving’ ability (*sensu* Borowiec, 2021; Swierk, 2019) is presumably a direct result of skin hydrophobicity or, rather, a hydrophobic skin is likely a necessary prerequisite for functional sustained rebreathing. Somewhat hastily, Boccia et al. (2021) have assumed that a hydrophobic skin would be a plesiomorphic trait of all anole lizards. Our findings nuance that notion. We find that while the key elements for hydrophobicity (i.e. spinulate skin surface) can be considered ancestral, the architecture and positioning of these elements has been changed, repeatedly and convergently, to meet the functionality of water repellency in semi-aquatic species. We believe our findings bring an additional dimension to the biological phenomenon described by Boccia et al. (2021); namely, that diving *Anolis* lizards not only repeatedly and independently evolved a specialized rebreathing behavior with the transitioning to a semi-aquatic lifestyle, but that its evolution presumably also coincided with, or was preceded by, the evolution of a highly hydrophobic structured skin to successfully do so. Additional data on the skin wettability properties from different body regions, particularly the head scales, would strengthen this argument.

Second, aside from facilitating plastron respiration, a hydrophobic structured skin surface may also save energy by reducing drag during underwater dives and swims by creating a low resistance air–water interface (Aljallis et al., 2013; Balasubramanian et al., 2004; Yu et al., 2020); a concept that has inspired the design of ship hulls (Busch et al., 2019). A third benefit is that, by generating a thermally insulating layer of air (Barthlott et al., 2016), a hydrophobic skin may slow down the cooling of the body in the water, allowing lizards to be active near optimal temperatures for a prolonged period of time. Water-shrews, for instance, can maintain a high body temperature while swimming in cold water because of the large amounts of air trapped in their waterproof fur (Vogel, 1990). Fourth, it may keep the skin dry when returning to land, thereby avoiding the added burden of extra weight from being wet. Basic rules of animal scaling (square-cube law) indicate that a non-wettable skin is particularly beneficial for small animals, such as lizards, as they may become too heavy (to move) otherwise. Reiterating a *Gedankenexperiment* by Haldane (1956), a man coming out of a bath carries with him a film of water that is about half a millimeter thick and weighs about half a kilogram; a wet mouse, however, has to carry its own weight in water, and a wet fly has to lift many times its own weight. Thus, a non-wettable skin is likely of critical importance for a small anole that regularly ventures into the water and less so for an anole that stays on land. Lastly, a hydrophobic skin may lower the extent of soiling by providing body-cleansing opportunities (Blossey, 2003; Liu and Jiang, 2012) as skin contaminants will easily wash away when lizards enter or leave the water. Surface hydrophobicity is known to facilitate self-cleaning in geckos (Hiller, 2009; Riedel et al., 2020; Spinner et al., 2013) and plants (Barthlott and Neinhuis, 1997; Neinhuis and Barthlott, 1997). Further empirical study is required to test these last four predictions.

### Convergence in *Anolis*

Semi-aquatic anoles have puzzled biologists for decades because of the apparent absence of a general ‘semi-aquatic bauplan’ (Leal et al., 2002; Muñoz et al., 2015) that would set them apart from the other ecomorph types onto which other *Anolis* lizards so famously have converged (Losos, 2009; Losos et al., 1998; Mahler et al., 2013). However, our findings provide support for convergent evolution of increased skin hydrophobicity, which implies that the apparent ‘lack of convergence in an otherwise convergent system’ (*sensu* Leal et al., 2002) may have resulted from an incomplete appreciation of the selective environment of semi-aquatic life. While traits associated with the locomotory system, such as limb length and sprint performance, are under strong selection in strict terrestrial anoles by the demands of moving and perching vertically on arboreal substrates (Feiner et al., 2020; Losos, 1990a,b, 2009), our study demonstrates that the major challenges faced by semi-aquatic species are likely imposed by the frequent and prolonged contact with water, promoting adaptive responses in the integumentary system. A more comprehensive study on skin form and function of anoles that incorporates species across the complete *Anolis* radiation, representing all ecomorphs, should be able to determine whether the enigmatic ‘aquatic’ ecomorph exists.

### Conclusion

Convergence of animal form and function can provide strong evidence for adaptive evolution by natural selection (Larson and Losos, 1996; Losos, 2011; Losos and Miles, 1994). We showed that the skin surface of semi-aquatic anole species was characterized by longer hair-like microstructures (spines and spinules) and a lower wettability in comparison to closely related terrestrial species. Moreover, our results revealed repeated evolution of increased skin hydrophobicity associated with the independent transitions to a semi-aquatic lifestyle. These findings support the spandrel hypothesis (Gould and Lewontin, 1979), which is that the pre-existing ornamented skin surface of anoles has acted as a facilitator or necessary precursor for the subsequent elaboration of a highly hydrophobic skin as an adaptation to semi-aquatic life. We also found that this spandrel has subsequently been rearranged repeatedly and convergently to meet the specific requirements of a hydrophobic skin. This is the first report of convergent evolution of hydrophobic structured skin as an adaptation to semi-aquatic life in a tetrapod radiation. This finding invites a new and exciting line of research about the ecological significance, evolutionary origin and developmental basis of hydrophobic skin surfaces in semi-aquatic anole lizards, which is crucial for understanding why and how the observed skin adaptations evolved in some and not other tetrapod lineages.

## Supplementary Material

Supplementary information
