# Supplementary material for: Convergent evolution of skin surface microarchitecture and increased skin hydrophobicity in semi-aquatic anole lizards
Source: J Exp Biol. 2021 Oct 14;224(19):jeb242939. doi: 10.1242/jeb.242939 (PMC8541734; doi:10.1242/jeb.242939)
Supplement: Supplementary information [file jexbio-224-242939-s1.pdf]

Table S1. List of museum specimens used in the current study, with information on the museum of collection, identification (ID) number, and snout-to-vent length (SVL).

Number	Species	Collection	ID number	SVL (mm)
1	*Anolis acutus*	MCZ	34764	61.91
2	*Anolis acutus*	MCZ	34759	63.71
3	*Anolis aquaticus*	MCZ	186135	74.69
4	*Anolis aquaticus*	MCZ	186143	60.85
5	*Anolis aquaticus*	UF	72360	68.2
6	*Anolis aquaticus*	UF	72371	66.25
7	*Anolis bartschi*	MCZ	93471	71.34
8	*Anolis bartschi*	MCZ	93460	70.73
9	*Anolis christophei*	MCZ	186676	44.31
10	*Anolis christophei*	MCZ	186680	46.57
11	*Anolis christophei*	UKansas	V33784	49.32
12	*Anolis christophei*	UKansas	V33782	49.24
13	*Anolis eugenegrahami*	MCZ	154497	72.63
14	*Anolis eugenegrahami*	MCZ	154507	67.94
15	*Anolis evermanni*	MCZ	36034	69.76
16	*Anolis evermanni*	MCZ	36000	71.69
17	*Anolis granuliceps*	MCZ	100364	50.69
18	*Anolis granuliceps*	MCZ	115695	45.79
19	*Anolis lionotus*	MCZ	19374	52.87
20	*Anolis lionotus*	MCZ	100432	51.89
21	*Anolis poecilopus*	MCZ	177829	57.74
22	*Anolis poecilopus*	MCZ	177830	65.89
23	*Anolis poecilopus*	UF	123004	69.24
24	*Anolis poecilopus*	UF	123017	67.42
25	*Anolis poecilopus*	UKansas	3355	60.83
26	*Anolis poecilopus*	UKansas	3356	62.84
27	*Anolis stratulus*	MCZ	R-179214	46.42
28	*Anolis stratulus*	MCZ	R-179222	44.39
29	*Anolis townsendi*	MCZ	R-139154	43.36
30	*Anolis townsendi*	MCZ	R-139148	46.53
31	*Anolis vermiculatus*	MCZ	38426	116.01
32	*Anolis vermiculatus*	MCZ	38428	120.76
33	*Anolis vermiculatus*	AMNH	R-78623	88.82
34	*Anolis vermiculatus*	AMNH	R-78627	117.32
35	*Anolis woodi*	MCZ	186125	60.66
36	*Anolis woodi*	MCZ	186177	68.78
37	*Anolis oxylophus*	MCZ	129353	69.65
38	*Anolis oxylophus*	MCZ	129351	61.38
39	*Anolis limifrons*	MCZ	109978	42.19
40	*Anolis limifrons*	MCZ	109976	42.25
41	*Anolis oculatus*	MCZ	60350	76.67
42	*Anolis oculatus*	MCZ	60353	75.53
43	*Anolis cuvieri*	MCZ	35975	122.89
44	*Anolis cuvieri*	MCZ	127119	115.95
45	*Anolis gracilipes*	MCZ	124408	56.91
46	*Anolis gracilipes*	MCZ	124405	51.94
47	*Anolis woodi*	LACM	148154	71.00
48	*Anolis woodi*	LACM	148168	81.53
49	*Anolis lionotus*	LACM	170282	78.16
50	*Anolis lionotus*	LACM	170285	79.86
51	*Anolis aquaticus*	LACM	166305	66.43
52	Anolis aquaticus	LACM	166325	68.59

Journal of Experimental Biology • Supplementary information
